# Radiological response of primary central nervous system lymphoma after corticosteroid therapy and its predictive value on overall survival: a multicenter study

**DOI:** 10.1007/s11060-026-05773-3

**Published:** 2026-08-29

**Authors:** Florian Scheichel, D. Pinggera, T. Rossmann, B. Popadic, S. Aspalter, V. Schön, A. Woehrer, M. M. Dorostkar, C. Dorfer, C. F. Freyschlag, F. Marhold

**Affiliations:** 1 Department of Neurosurgery, University Hospital St. Pölten – NOE LGA, Karl Landsteiner University, Dunant-Platz 1, St. Pölten, 3100 Austria; 2 Department of Neurosurgery, Medical University Innsbruck, Innsbruck, Austria; 3 Department of Neurosurgery, Neuromed Campus, Kepler University Hospital, Linz, Austria; 4 Institute of Neuropathology and Neuro-Molecular-Pathology, Medical University, Innsbruck, Austria; 5 Institute of Clinical Pathology and Molecular Pathology of the Lower Austria Central Region, University Hospitals St. Pölten and Krems - NOE LGA, Karl Landsteiner University, St. Pölten, Austria

**Keywords:** Primary Large-B-Cell Lymphoma of the CNS, Corticosteroid therapy, Overall survival

## Abstract

**Background:**

Primary Large-B-Cell Lymphoma of the CNS (PCNS-LBCL) is a rare, aggressive tumor often sensitive to corticosteroid therapy (CST). This multicenter study investigated the spectrum of radiological responses to routine CST and evaluated its impact on patient prognosis.

**Methods:**

The researchers utilized a prospective cohort of 18 patients for volumetric MRI analysis and a combined prospective-retrospective cohort of 31 patients for 2D tumor analysis. Patients received CST post-biopsy, with follow-up MRIs performed on the seventh day (median) after surgery. The study correlated radiological responses with CST dosage, LDH levels, and overall survival (OS) using Kaplan-Meier and Firth-corrected Cox regression.

**Results:**

In the prospective cohort, 83.3% of tumors regressed with a median regression of 40%, while 16.7% progressed despite CST resulting in a median progression of 46%. These changes were not dose-dependent, but there were indications of a correlation with LDH levels. Patients showing regression had a median OS of 31.4 months, compared to 3.9 months for tumors progressing during CST (*p* = 0.015). Firth-corrected Cox regression showed, despite the small sample size, that radiological response (*p* = 0.02) could be a promising prognostic factor in a model including age, type of therapy and Karnofsky performance status.

**Conclusion:**

This study provides the first structured description of the range of radiological responses of PCNS-LBCL to CST. Furthermore, we found exploratory indications that radiological response to CST may predict outcome indicating shared sensitivity to other cytotoxic drugs. Further studies are necessary to fully understand diagnostic and prognostic implications of CST sensitivity in PCNS-LBCL and its underlying mechanisms.

## Background

Primary large B-cell lymphoma of the CNS, also known as primary central nervous system lymphoma (PCNS-LBCL) is a rare disease accounting for approximately 3% of all intracranial tumors [1, 2]. Surgery is generally limited to gaining tissue for establishing the histological diagnosis via biopsy [3, 4]. PCNS-LBCL cells however can be highly sensitive to corticosteroid therapy (CST) potentially reacting with distinct shrinkage of contrast-enhancing tumor in MRI [5–7]. Accordingly, CST pre-treated PCNS-LBCL may show subtotal or total loss of neoplastic B-cells [8, 9], potentially leading to inconclusive biopsies. Therefore, if clinically possible, CST should be avoided until diagnostic tissue has been acquired [10]. However, preoperative CST is often administered initially due to the patients’ presentation with neurological symptoms and prior to consultation of a neurooncological center. While regression is typical, the response pattern of PCNS-LBCL can be highly heterogenous and ranges from vanishing of the tumor to progression despite CST in some cases. Up to date, description of radiological responses is limited to retrospective studies with inhomogeneous data regarding CST dosage, duration and timing of pause [11–13]. After initial tumor regression following CST, most tumors recur after a short period of time, meaning that CST alone is not a viable treatment option. However, long-lasting remissions have been reported in a few cases [14–16]. The patient-level phenotypic heterogeneity in radiological and clinical response has not yet been investigated in terms of its prognostic implications.

The aim of the present multicenter prospective study was to perform a comprehensive analysis of the radiological changes that can be expected after routinely administered CST doses and its possible influence on clinical outcome.

## Methods

### Prospective volumetric cohort

We performed a multicenter prospective study including patients with suspected PCNS-LBCL that were planned for stereotactic or endoscopic biopsy in three neurosurgical centers in Austria (University Hospital St. Poelten – Karl Landsteiner University Krems, Medical University Innsbruck, Neuromed Campus - Kepler University Hospital - Linz) after signing informed consent. We excluded patients that showed signs of systemic lymphoma by means of a staging-CT or bone-marrow biopsy, that were immunocompromised or positive for Epstein Barr virus upon EBER in situ hybridization. Biopsy had to show a Primary Large-B-Cell Lymphoma of the CNS (PCNS-LBCL). After surgery, all participating centers routinely administered CST to alleviate neurological symptoms following comparable protocols, involving an initial bolus of 40 mg of dexamethasone followed by 16 mg daily postoperatively, with the dose halved every 3 days. We planned an additional thin-slice MRI 5 to 10 days after surgery for comparison with the preoperative navigational MRI, though deviations of the protocol were allowed to not lose patients due to availability of MRI. Patients were, however, excluded if the second MRI would have delayed the definitive treatment.

Using 3D Slicer [17], we performed volumetric analysis of contrast-enhancing lesions on both MRIs. Relative tumor volume changes were correlated with preoperative tumor volume, cumulative CST dosage, dose per body surface area (BSA), CST duration, and LDH.

The study was approved by the local ethics committees of the participating centers and was conducted according to the ethical standards as laid down in the 1964 Declaration of Helsinki and its later amendments or comparable ethical standards.

### Combined retrospective and prospective cohort

A secondary analysis was conducted leveraging the prospective cohort described above and an additionally retrospective cohort of patients previously published in 2021 [12] by our group. We included all patients treated either at the University Hospital St. Pölten or Medical University Innsbruck, that had undergone at least two preoperative MRIs with corticosteroid therapy between those MRIs and in whom detailed corticosteroid data were available. Furthermore, it was necessary that corticosteroid therapy was initiated not more than 2 days prior to the first MRI and was ongoing or not significantly paused before the second MRI to rule out regression prior to the first MRI or progression after an initial regression. We then calculated the largest cross-sectional area of the contrast enhancing tumor in an axial 2D-slice for all patients (retrospective and prospective). We performed the same analysis for prospective patients to have a consistent measurement for the entire cohort; however, this step was done blinded for the volumetric result. With the complete cohort we again tested for predictive variables of radiological dynamic. We furthermore performed a survival analysis to investigate if the response pattern of PCNS-LBCL on CST divided into progression or regression, defined as any decrease or increase, respectively, of the largest cross-sectional tumor area, has predictive value for overall survival. After Kaplan Meier analysis we then performed univariable and consequently multivariable Firth-corrected Cox regression including the response pattern on CST, age, Karnofsky performance status scale and type of therapy (including systemic high-dose methotrexate or not) looking for significant independent prognostic factors. We did not add further factors due to the limited numbers of patients and events.

### Statistical analysis

Statistical analyses were performed using SPSS v30 (IBM Corp.) and R v4.4.3. Normality was assessed via the Kolmogorov-Smirnov test. Data are presented as mean ± SD, median (range), or frequencies (percentages). Group comparisons and correlations were calculated using independent t-tests, and Pearson (normally distributed data) or Kendall-tau-b (skewed data) correlations, respectively. Agreement between the relative volumetric and 2D responses was assessed using a two-way mixed, absolute-agreement, single-measures intraclass correlation coefficient (ICC). Survival was analyzed using the Kaplan-Meier method and univariable/multivariable Firth-corrected Cox regression. Significance was set at *p* < 0.05.

## Results

A total of 40 patients were evaluated for eligibility for the study. We had to exclude 13 patients because a second MRI scan was not performed in order not to delay definitive treatment; 4 patients had a final integrated diagnosis of glioblastoma, 3 patients had systemic lymphoma; one patient was excluded due to insufficient MRI quality, and one patient was immunocompromised. A total of 18 patients were thus enrolled in the prospective study. Ten patients were female (55.6%) and eight were male (44.4%). Three patients had already been receiving CST before the biopsy. In two of these cases, CST had been interrupted for about one week before surgery and in one patient CST was continued until biopsy. One patient underwent an endoscopic biopsy of a mass in the third ventricle; in all other patients a stereotactic biopsy was performed. There were no surgical complications, particularly no postoperative hemorrhage that would have compromised the volume analysis.

Patients had a median time to second MRI and duration of postoperative CST until postoperative MRI of 7 days (range 6–13, 5 patients with 6 days, 7 patients with 7 days, 2 patients with 8 days, and 1 patient each with 9, 10, 11 and 13 days) and a median cumulative postoperative dose of 112 mg dexamethasone (48–140 mg). Apart from one patient each with 48 mg and 86 mg, all patients received between 104 and 140 mg.

**Fig. 1:**
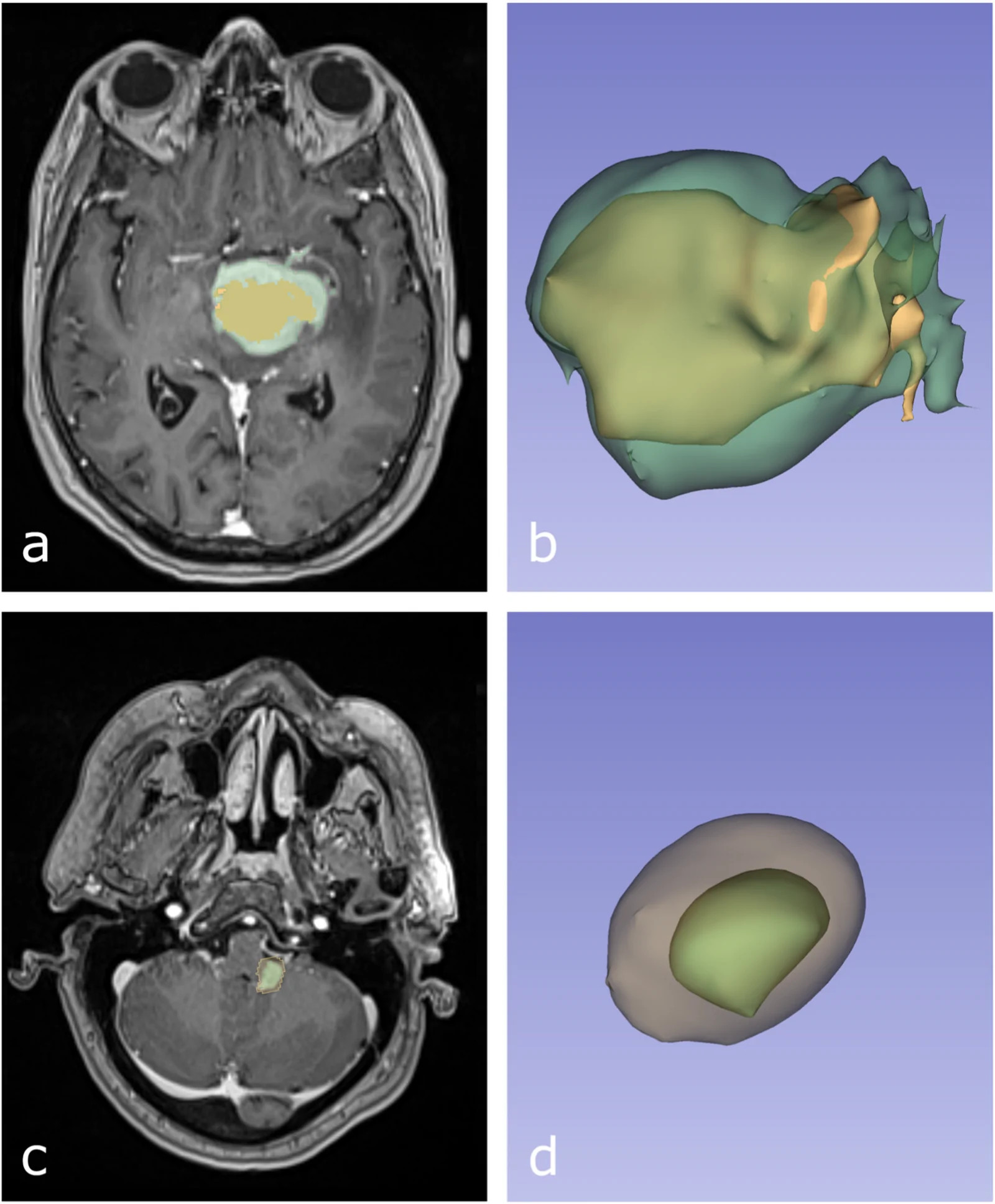
Volumetric analysis of tumor volume; the upper images show a case in which the disease progressed despite CST, while the lower images show regression following CST. a and c: post-CST axial slice of T1 MRI with contrast enhancement showing the tumor after CST. Yellow marks the corresponding preoperative lesion before corticosteroids and green the tumor after CST. b and d: volumetric rendering of preoperative and post-CST tumor

We observed a radiological regression after CST in 83.3% of cases with a median regression of tumor volume of 40% (range 77% to 1%). Progression was noted in 16.7% with a median radiological progression 46% (range 7% to 94%). Examples of typical regression and progression are given in Fig. 1.

Volumetric analysis revealed a mean tumor volume before surgery and after CST of 16.1 cm^3^ (SD = 14.4 cm^3^) and 11.9 cm^3^ (SD = 10.7 cm^3^) respectively.

Our data provided no evidence that changes in PCNS-LBCL volume following CST were dose dependent (𝛕=0.072, *p* = 0.694, 95%-CI=-0.27–0.40) or dependent on the duration of CST which corresponds to the duration until postoperative MRI (𝛕=0.234, *p* = 0.255, 95%-CI=-0.15–0.56). The median cumulative dose per m^2^ body surface area was 56.9 mg, ranging from 27.18 mg to 73.45 mg, and correlated with the duration of CST (𝛕=0.510, *p* < 0.009, 95%-CI = 0.21–0.72) as the most common regimen consisted of 40 mg of dexamethasone postoperatively and then 16 mg over three days, followed by gradual reduction in dosage every three days.

Of the three patients with CST prior to surgery, the one patient with persistent CST following a postoperative dose increase showed a 40% reduction in tumor volume; the two patients who underwent a pause of CST prior to biopsy showed either no response (a 1% reduction) or distinct progression of 46%. We performed a sensitivity analysis after excluding these patients; the remaining 15 patients showed regression in 86.7% (with a median regression of 42.3%) and progression in 13.3% (with a median progression of 50.8%).

Our prospective data suggest a potential correlation between preoperative LDH and radiological response after CST treatment (*r* = 0.417, *p* = 0.085, 95%-CI=-0.06–0.74). Preoperative LDH levels, however, did not correlate with preoperative tumor volume (*r*=-0.178, *p* = 0.48, 95%-CI = -0.595–0.315).

### Combined retrospective and prospective two-dimensional analysis

**Fig. 2:**
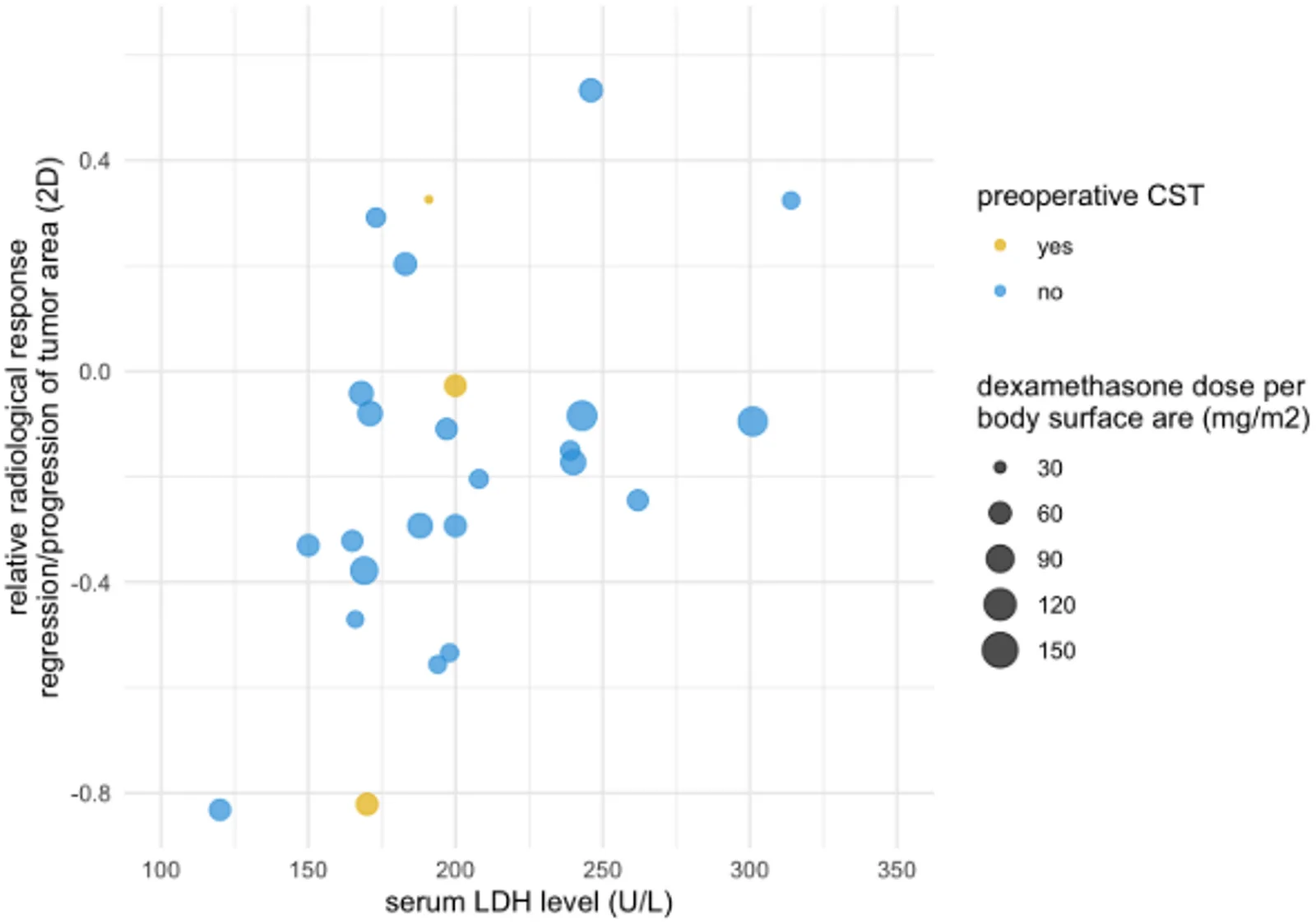
Correlation of LDH and relative radiological response of PCNS-LBCL due to CST

We searched our available retrospective cohort for patients treated in the participating centers with at least two preoperative MRI scans with intervening CST. Of 143 patients, we included 13 retrospective cases, resulting in a combined retro- and prospective cohort of 31 patients. Overall, we observed regression of the 2D tumor area in the axial MRI slice in 74.2% (*n* = 23) of cases, and progression in 25.8% (*n* = 8). In all cases of the prospective cohort, the assessment of whether the tumor had regressed or progressed was consistent between the volumetric and 2D assessments and both methods showed comparable relative responses (ICC = 0.87, 95%-CI = 0.67–0.95, *p* = 0.001). In the combined cohort, the correlation between LDH and the quantitative reduction in the largest tumor area was more pronounced (𝛕 = 0.317, *p* = 0.026, 95%-CI = 0.05–0.55, Fig. 2).

### Correlation of MRI response to overall survival

We then divided the cohort into two groups based on whether the PCNS-LBCL showed regression or progression following CST in the 2D analysis. The patients were comparable in terms of known prognostic factors such as mean age, type of therapy, MSKCC, and involvement of deep brain structures (Table 1). Preoperative tumor size was also comparable, with a mean maximum tumor area of 9.22 cm² and 9.0 cm² in patients with regression and progression, respectively.

**Fig. 3:**
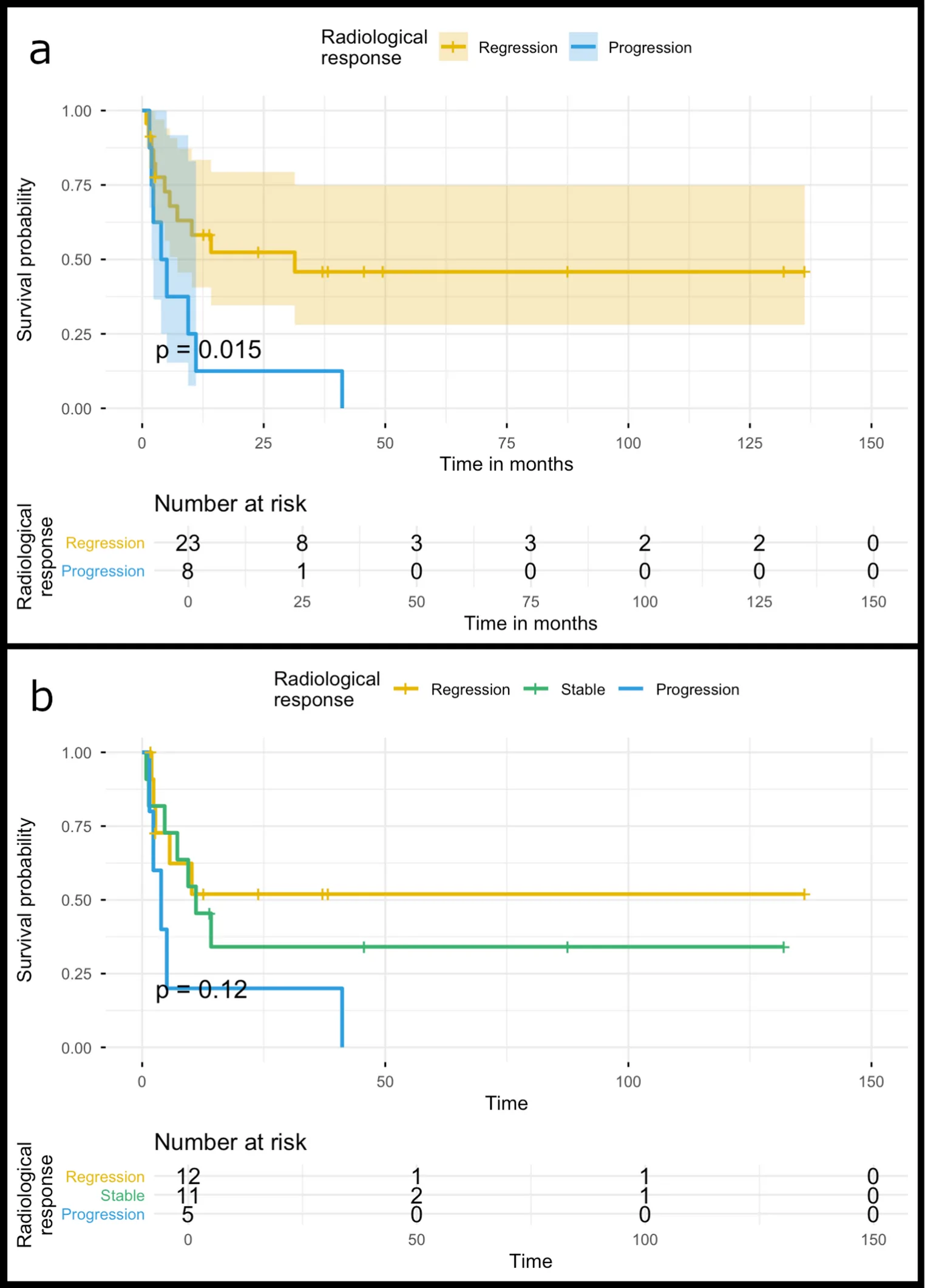
Kaplan-Meier curve analysis showing a significantly better survival rate in patients with disease regression following CST compared to patients with disease progression despite CST (**a**). A distinct trend consistent with our primary analysis was observed in a quantitative subgroup analysis based on RECIST criteria defined as regression of at least 30%, stable disease, or progression of at least 20%; but these differences were not statistically significant (**b**)

Overall survival was significantly longer in patients who experienced regression of PCNS-LBCL following CST (31.4 months) than in patients who experienced progression despite CST (3.9 months; Kaplan-Meier analysis, *p* = 0.015, Fig. 3 - a). Since three patients had received CST previously, we performed a sensitivity analysis excluding these three pretreated cases. In this adjusted cohort, the survival disadvantage for patients with MRI progression remained statistically significant (median OS 3.9 months with progression, median OS not reached for patients with radiological regression; *p* = 0.023).

A sensitivity analysis was performed with patients stratified using thresholds of radiological response (based on RECIST criteria [18]) of more than 30% regression, stable disease, and more than 20% progression. Although limited by the small sample size, the data still showed a distinct trend consistent with our primary analysis with median survival not reached for regression, 11.1 months for stable disease and 3.9 months for progression, respectively (*p* = 0.125, Fig. 3 - b). Table 2 shows univariable regression of known predictors of survival in PCNS-LBCL and radiological response to CST. Age, KPS of at least 70, type of induction therapy (containing high-dose MTX or not) and radiological response showed promising results with p-values below 0.1 and were included in a multivariable Firth-corrected Cox regression. This multivariable analysis showed that radiological response of PCNS-LBCL following CST was associated with a hazard ratio of 3.37 in the event of tumor progression despite CST in our dataset (*p* = 0.02, 95%-CI = 1.21–9.35, Table 3).

**Table 1:** Both groups were comparable in regard of known predictors of overall survival and preoperative tumor volume

	Radiological response		
	Regression	Progression	
**Mean age**	68.6 years	66.1 years	*P* = 0.508
**Therapy**			*p* = 0.276
MATRix	11 (47.8%)	3 (37.5%)	
MTX, Cytarabin, Rituximab	2 (8.7%)	0	
MTX, Rituximab	5 (21.7%)	1 (12.5%)	
MTX, Cytarabine	0	2 (25%)	
MTX	2 (8.7%)	0	
Radiation and MTX	1 (4.3%)	0	
Whole brain radiation	1 (4.3%)	1 (12.5%)	
Intrathecal MTX	1 (4.3%)	1 (12.5%)	
**MSKCC**			*p* = 0.744
Class 1	1 (4.3%)	0 (0%)	
Class 2	17 (73.9%)	7 (87.5%)	
Class 3	5 (21.7%)	1 (12.5%)	
**Involvement of deep brain structures**			*p* = 0.66
Yes	17 (73.9%)	5 (62.5%)	
No	6 (26.1%)	3 (37.5%)	
**Mean Pre-OP Tumor**	9.22 cm^2^ (SD 7.6 cm^2^)	9.00 cm^2^ (SD 6.1 cm^2^)	*p* = 0.946

**Table 2:** Univariable regression of known predictor variables and radiological tumor response

	Hazard ratio	95%-CI	*p*
KPS < 70	2.497	0.893–6.969	0.081
Age	1.05	0.995–1.109	0.077
LDH	0.996	0.984–1.008	0.532
Radiological progression despite CST	2.979	1.181–7.51	0.021
MSKCC (Class2)	0.810	0.105–6.257	0.84
MSKCC (Class3)	2.049	0.235–17.861	0.516
Type of induction therapy*	7.139	1.663–30.657	0.008

* - Hazard ratio for patients not receiving a treatment regimen containing systemic high-dose methotrexate. MSKCC stands for Memorial Sloan-Kettering Cancer Center score [19]

**Table 3:** Multivariable Firth corrected cox regression

	Hazard ratio	95%-CI	*p*
Age	1.05	0.98–1.13	0.141
KPS < 70	1.09	0.34–3.52	0.885
Type of induction therapy*	3.94	0.85–18.15	0.079
Radiological progression despite CST	3.37	1.21–9.35	0.020

* - Hazard ratio for patients not receiving a treatment regimen containing systemic high-dose methotrexate

## Discussion

Preoperative CST can complicate the diagnostic evaluation of PCNS-LBCL, as histological changes—including the complete disappearance of malignant B-cells—have been reported in up to 52% of cases [8, 9]. Clinically, threefold increases of inconclusive biopsies have been described after preoperative CST with different doses and periods of CST interruption [12, 20, 21]. Radiological regression of contrast enhancing tumor is often used as surrogate parameter for response to CST and the likelihood of inconclusive biopsy [22]. However, this is the first study to thoroughly investigate radiological response patterns to CST, their influencing factors and potential prognostic value.

Available data vary immensely, most likely due to the underlying heterogeneity of CST sensitivity itself but also due to the inhomogeneous available data. Porter et al. described 55 patients with serial preoperative computed tomography or MRI, of whom 49 (89.1%) showed no significant change upon CST, three regression (one a vanishing tumor) and three progression [11]. Bullis et al described 4 patients with MRIs before and after CST and all of them showed regression after CST [13]. In our previous study, 23.4% of 64 patients showed regression following CST, 18.8% showed no change and tumor progression occurred in 57.8%. [12] However, these numbers may be skewed, as the scientific focus of these retrospective studies was on the success of the biopsy. CST was administered prior to surgery and was most likely being discontinued immediately if PCNS-LBCL was strongly suspected in most patients which may have led to progression following a possible prior regression. On the other hand, some patients may have required prolonged CST due to symptoms until the second imaging. Therefore, due to inconsistent data, no definite conclusions can be drawn regarding the distribution or extent of radiological response to CST.

In the present study we sought to establish a comparable group of patients with MRI scans performed before and after CST. We therefore used the period following the biopsy and prior to the start of PCNS-LBCL-directed induction treatment—during which tissue had already been acquired for diagnosis and patients were receiving CST to alleviate neurological symptoms—to perform a second MRI scan for comparison. Our prospective cohort showed that most patients experienced regression of the contrast enhancing tumor (83.3%) and only 3 patients showed progression despite CST (16.7%). There was a wide range from 77% reduction in tumor volume to 94% progression despite CST. This is of interest because it demonstrates that, for unknown reasons, the sensitivity of PCNS-LBCL to CST varies distinctively from patient to patient. Our data did not provide any evidence that these differences were due to initial tumor volume, cumulative dose of CST, cumulative dose per body surface area or the duration of CST. There were some indications that patients with higher preoperative serum LDH levels were more prone to progression despite CST. Elevated LDH levels are a known adverse prognostic factor for systemic lymphomas and PCNS-LBCL [23]. Elevated LDH levels could therefore indicate a more aggressive tumor, which in turn may respond less effectively to CST and other treatments.

The effect of CST, whether significant or minimal, is generally only temporary and regrowth of malignant B-cells and progression is inevitable in most cases. CST is therefore not used or recommended as stand-alone treatment. However, a small number of historic case reports and case series describe prolonged remission of PCNS-LBCL after solely CST which may be due to extreme CST sensitivity. [14–16, 24] We therefore sought to investigate whether CST sensitivity could be a prognostic predictor in patients with PCNS-LBCL. To this end, we evaluated MRI findings before and after CST using two-dimensional analysis in 31 patients; 74.2% showed regression and 25.8% progression. Patients with regression had a statistically significant longer median overall survival compared to those with progression despite CST (31.4 months vs. 3.9 months). In a multivariable Cox regression analysis, radiological response pattern of PCNS-LBCL following CST was a promising factor reaching statistical significance in our data set. However, due to the small sample size and the limited number of events, we were unable to include all known predictive variables; therefore, our results should be considered exploratory.

To date, radiological regression after CST has been investigated in multiple studies for influence on diagnostic yield if administered before biopsy. To the best of our knowledge, our data demonstrate for the first time that radiological response patterns to CST might also have prognostic implications. PCNS-LBCL lesions that show progression despite CST may be more aggressive and drug resistant [25]. To date it remains unclear what determines CST sensitivity in PCNS-LBCL and tumor biological features of malignant clones may play a role. Understanding these mechanisms would be important for a better assessment of prognosis and, consequently, for a potential further subclassification of PCNS-LBCL.

### Limitations

1. All prospective patients underwent stereotactic or endoscopic biopsy. The amount of tissue removed due to the surgery is small. However, in cases of initially small lesions the relative amount of removed tissue is more significant than in larger tumors and might therefore skew our volumetric data. While we acknowledge the resulting limitation, we argue that this is the only method to study the CST-sensitivity in patients with histologically confirmed PCNS-LBCL, as preoperative CST would increase the risk of inconclusive biopsy.

2. Due to the small cohort, our exploratory results require validation through prospective multicenter studies to address confounders.

3. For the survival analysis we had to use retrospective data due to the limited number of patients and events in the prospective data set. As described in the methods section, we only included retrospective patients with two preoperative MRIs and CST in the timespan between those studies to increase data quality.

4. We chose overall survival as the more reliable metric over progression-free survival due to extended study period and heterogeneous follow-up strategies. We also tried to analyze salvage therapies, but adjuvant treatment at other hospitals and inconsistent documentation precluded analysis. Possible confounders like treatment heterogeneity, salvage therapies and modest sample size must therefore be considered when interpreting results.

## Conclusion

The study provides the first structured description of the range of radiological responses of PCNS-LBCL to perioperative CST. Lower LDH levels prior to the initiation of CST might correlate with radiologically regressive lesions. Our study provided initial, exploratory indication that progression of PCNS-LBCL despite CST could potentially be a negative predictor of overall survival, putatively indicating overall worse sensitivity to MTX and other commonly administered drugs. Further studies are necessary to validate our results and subsequently understand the diagnostic and prognostic implications of CST sensitivity in PCNS-LBCL and its underlying mechanisms.

## Data Availability

The datasets analyzed during the current study are available from the corresponding author on reasonable request.
